# The evolutionary history of Brachyury genes in Hydrozoa involves duplications, divergence, and neofunctionalization

**DOI:** 10.1038/s41598-023-35979-8

**Published:** 2023-06-09

**Authors:** Alexandra A. Vetrova, Daria M. Kupaeva, Alena Kizenko, Tatiana S. Lebedeva, Peter Walentek, Nikoloz Tsikolia, Stanislav V. Kremnyov

**Affiliations:** 1 Laboratory of Morphogenesis Evolution, Koltzov Institute of Developmental Biology RAS, Vavilova 26, Moscow, 119334 Russia; 2 Department of Embryology, Faculty of Biology, Lomonosov Moscow State University, Leninskiye Gory 1/12, Moscow, 119234 Russia; 3 Institute of Science and Technology Austria (ISTA), Am Campus 1, 3400 Klosterneuburg, Austria; 4 Department for Molecular Evolution and Development, Centre of Organismal Systems Biology, University of Vienna, Althanstraße 14, 1090 Vienna, Austria; 5 Renal Division, Internal Medicine IV, Medical Center, Faculty of Medicine, University of Freiburg, 79106 Freiburg, Germany; 6 CIBSS-Centre for Integrative Biological Signalling Studies, University of Freiburg, 79104 Freiburg, Germany; 7 Institute of Anatomy and Embryology, University Medical Center Göttingen, Kreuzbergring 36, 37085 Göttingen, Germany

**Keywords:** Evolutionary developmental biology, Zoology

## Abstract

Brachyury, a member of T-box gene family, is widely known for its major role in mesoderm specification in bilaterians. It is also present in non-bilaterian metazoans, such as cnidarians, where it acts as a component of an axial patterning system. In this study, we present a phylogenetic analysis of Brachyury genes within phylum Cnidaria, investigate differential expression and address a functional framework of Brachyury paralogs in hydrozoan *Dynamena pumila*. Our analysis indicates two duplication events of Brachyury within the cnidarian lineage. The first duplication likely appeared in the medusozoan ancestor, resulting in two copies in medusozoans, while the second duplication arose in the hydrozoan ancestor, resulting in three copies in hydrozoans. Brachyury1 and 2 display a conservative expression pattern marking the oral pole of the body axis in *D. pumila*. On the contrary, Brachyury3 expression was detected in scattered presumably nerve cells of the *D. pumila* larva. Pharmacological modulations indicated that Brachyury3 is not under regulation of cWnt signaling in contrast to the other two Brachyury genes. Divergence in expression patterns and regulation suggest neofunctionalization of Brachyury3 in hydrozoans.

## Introduction

Brachyury (or T) is a founding member of T-box transcription factor family^1^ first identified in a mutant mouse strain^2^. Mice lacking one allele of Brachyury exhibit a short-tail phenotype^3^, while the prenatal lethal loss of both alleles leads to severe deficiencies in mesoderm and axial structure formation^4,5^. Subsequent studies demonstrated that Brachyury is highly conserved and present not only in chordates, but in most metazoan animals ranging from ctenophores to sea urchins, as well as in ichthyosporeans, filastereans, and several early-branching fungi^6–9^.

Brachyury plays a crucial role in notochord formation in various chordates (reviewed in ^10^) and mesoderm specification in bilateria in general (reviewed in ^11^), and its evolutionary primary function is possibly associated with germ layer demarcation and morphogenesis during gastrulation^12,13^. It is also an important component of the axial patterning gene regulatory network^14^.

Though functions of Brachyury were examined in a limited number of species, patterns of its expression during embryonic development are well studied. One of the Brachyury expression domains is conservatively detected at one pole of the body axis (e. g., oral pole in cnidarians, posterior pole in deuterostomes)^15–18^, where the site of cell internalization is also located in the gastrulae of most animals. Within hydrozoans, Brachyury expression was demonstrated in the site of cell ingression during gastrulation in the embryos of *Clytia hemisphaerica*^19^. Brachyury is expressed around a blastopore in ctenophores^8^, anthozoans^12,20^, echinoderms^21,22^, amphioxus^23^, and all vertebrates investigated so far (reviewed in ^10^), though this expression domain was lost in ascidians^24^. In annelids, mollusks, and insects, Brachyury expression is also associated with the blastopore, though this expression domain dissolves to various degrees (reviewed in ^11^).

A single copy of the Brachyury gene is present in genomes of most Metazoans. However, there are several exceptions. Within chordates, *Xenopus laevis* has four Brachyury genes ^25,26^, where tbxt.L/tbxt.S (Xbra and Xbra2) and tbxt.2.L/tbxt.2.S (Xbra3) each are considered to be alloalleles, arising from the recent genome duplication^25–27^. *X. tropicalis* contains two Brachyury genes, one of which is clustered with Xbra/Xbra2 (tbxt) and the other corresponds to Xbra3 gene of *X. laevis* (tbxt.2)^28^. Teleost fish, such as medaka, zebrafish and three-spined stickleback, possess two Brachyury genes (Bra and Ntl) in their genomes^28,29^. Brachyury is present in two copies in the basal chordate amphioxus^23,30,31^. According to phylogenetic analysis, duplication events occurred not in the chordate ancestor, but in all three chordate lineages independently^28,32^. Among non-chordate metazoans, the hydrozoans *Hydra* and *C. hemisphaerica* have at least two copies of the Brachyury gene^13,33^.

Thorough phylogenetic analysis is required to understand the evolution of Brachyury genes within cnidarians, in particular, whether gene duplication occurred in the common hydrozoan ancestor or if there were several independent lineage-specific events. To resolve this issue, we aimed to reconstruct the phylogenetic tree of Brachyury genes within phylum Cnidaria. Our data indicate a first gene duplication in the common ancestor of Meduzozoa. Strikingly, Brachyury has undergone one more duplication in the hydrozoan lineage, where we found three paralogs of Brachyury in most species. Next, analysis of gene expression patterns of Brachyury paralogs in the hydrozoan *Dynamena pumila* during normal development and in the colony demonstrated very different expression dynamics of DpBra3 from the expression of DpBra1 and DpBra2. Since it is known that Brachyury is a direct target gene of the cWnt pathway^33,34^, we tested, if all three Brachyury paralogs are still under regulation of cWnt signaling. Data obtained from pharmacological modulations demonstrate that DpBra3 is differently regulated in comparison with DpBra1 and DpBra2. Taken together, our results suggest that the duplication of Brachyury genes resulted in the neofunctionalization of the Brachyury3 in the hydrozoan lineage.

## Results

### Diversity and phylogeny of cnidarian brachyury genes

To address the evolution of the Brachyury gene family within cnidarians, first we conducted TBLASTX search of the previously published transcriptome of *D. pumila*^35^ with the published *C. hemisphaerica* Brachyury gene sequences as an initial query. We recovered three sequences of Brachyury-like genes from the *D. pumila* transcriptome and used them as queries for TBLASTX searches against ten more medusozoan transcriptomes (see “Methods”). Together with four already known anthozoan sequences, a total of 33 Brachyury sequences were identified from 16 Cnidaria species.

**Figure 1:**
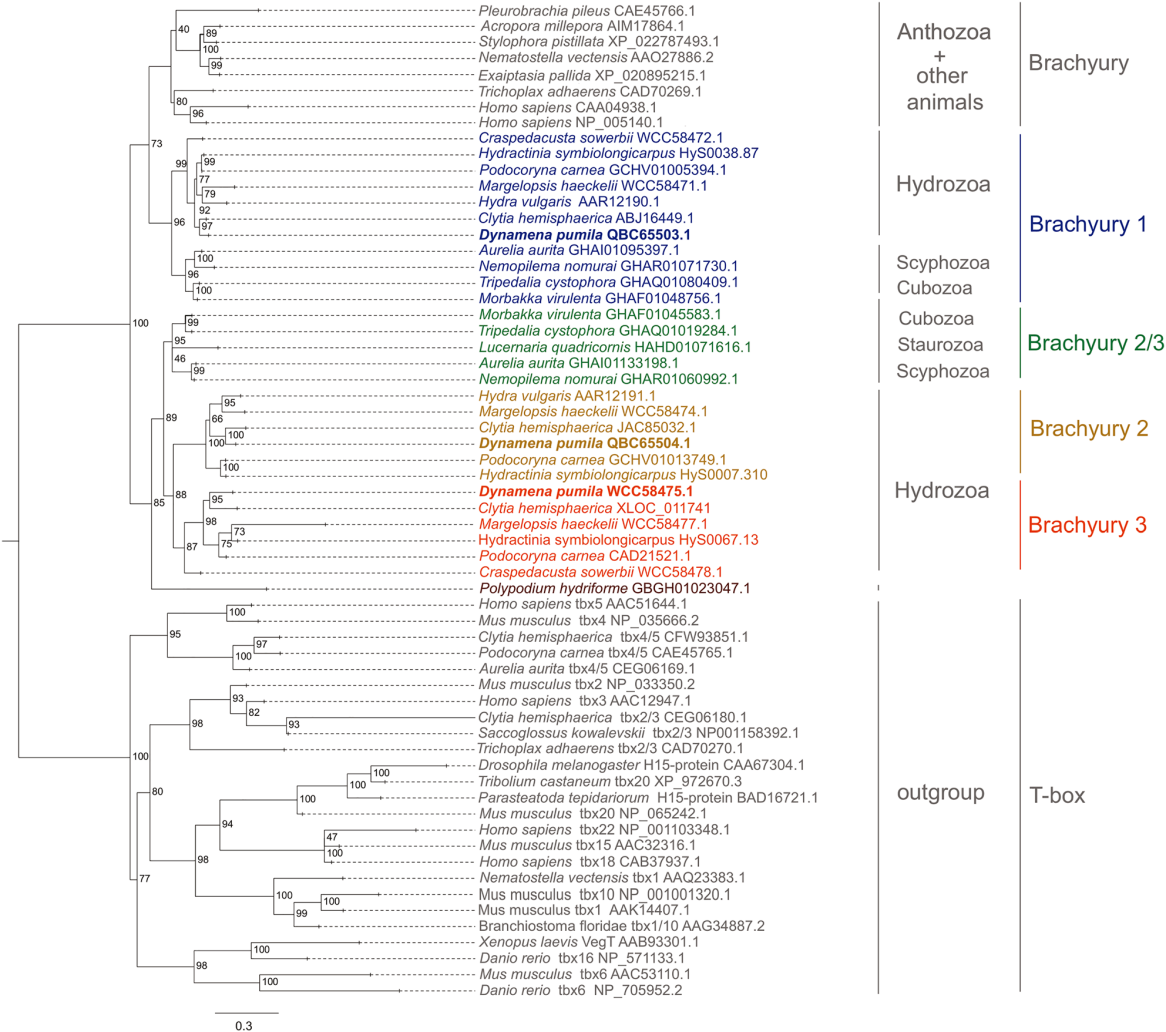
ML phylogenetic tree of Brachyury family members, rooted with TBX genes. Numbers at nodes are bootstrap values, shown as percentages. The scale indicates expected amino acid substitution per site. *D. pumila* genes are in bold.

A maximum likelihood tree was generated using translated amino acid sequences with the best-fit JTT++ R5 model (Fig. 1). For this analysis, a total of 41 Brachyury sequences were used representing all major metazoan groups except Porifera. Since the T-box transcription factor family includes classes of Tbx genes besides Brachyury^9^, sequences of metazoan Tbx genes were used as an out-group to root the tree. To test the robustness of the tree topology, we also used the conservative T-box domain for the alignment. Additional maximum likelihood tree with the same overall topology was generated using only T-boxes of analyzed sequences (Supplementary Information Fig. S1).

All analyzed anthozoan species possess a single Brachyury gene (Fig. 1). However, our transcriptomic survey revealed more Brachyury genes within Medusozoa besides Brachyury1 (Fig. 1). Medusozoan-only Brachyury genes belong to cubozoan, scyphozoan and hydrozoan clades. They cluster together with the Brachyury/Brachyury1 genes with a high nodal support (100% bootstrap value). Cubozoan and scyphozoan Brachyury2/3 genes cluster together, and hydrozoan-only Brachyury genes form a sister group to them. In turn, hydrozoan-only Brachyury genes include two sister groups, Brachyury2 and Brachyury3, with a well-supported bootstrap value (88%) (Fig. 1). Interestingly, a previously studied Brachyury transcript (AJ428494.1) of *Podocoryne carnea*^36^ is orthologous to Brachyury3 according to our analysis. Thus, all analyzed hydrozoan species have three Brachyury genes, with the exception of *Craspedacusta sowerbii* and *Hydra vulgaris*, which lack Brachyury2 and Brachyury3, respectively.

For a more thorough analysis of Brachyury genes in *Hydra* we searched for Brachyury transcripts in gene models of Hydra 2.0 (*Hydra magnipapillata*)^37^ and HydraAEP (*Hydra vulgaris*)^38^ genome assemblies with modified PIA3 pipeline, which allowed us to retrieve T-box protein class information automatically. This analysis confirmed that *Hydra* genomes contain only two Brachyury genes and several T-box genes (see an example of tree output in Supplementary Information Fig. S2). The manual search also retrieved only two Brachyury genes in *Hydra*. All these results increase the likelihood that *Hydra* indeed lost Brachyury3.

### Comparison of sequence conservation of hydrozoan Brachyury proteins

**Figure 2:**
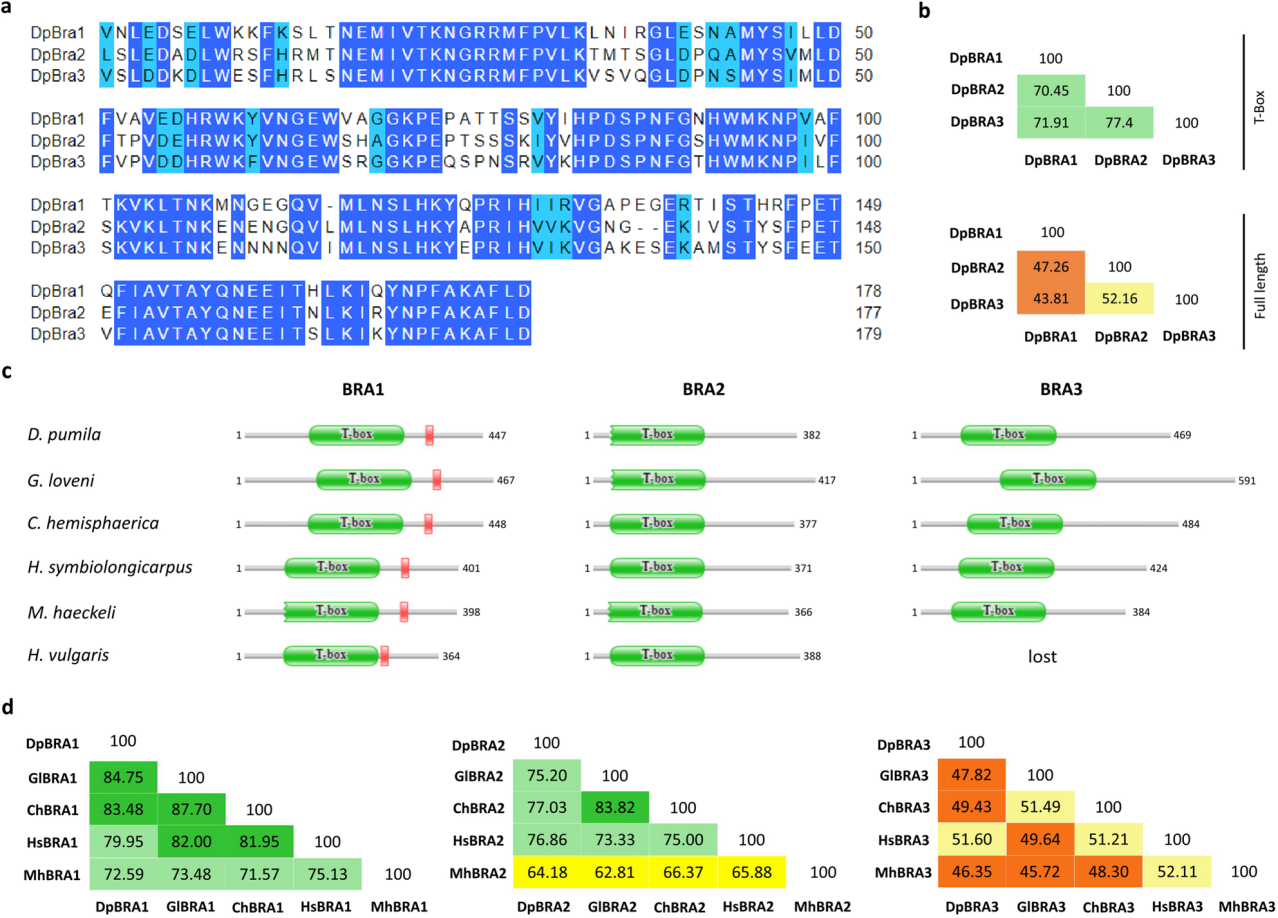
Comparison of sequence conservation among deduced hydrozoan Brachyury proteins. (**a**) Alignment of T-boxes of *D. pumila* Brachyury proteins. Amino acid identities are blue, light blue indicates that the residue is not identical but at least similar to the column consensus. (**b**) Percent identity matrix of T-boxes and full length Brachyury proteins in *D. pumila*. Colors here and below represent bands of percent identity: orange, 40–49%; light yellow, 50–59%; bright yellow, 60–69%; mint, 70–79%; green, 80–89%. (**c**) Domain architecture of predicted hydrozoan Brachyury proteins. Red box corresponds to R1 repressor domain. A jagged edge indicates that a sequence match does not match the full length of the HMM that models a pfam entry. Numbers indicate the length of the protein in amino acids. (**d**) Percent identity matrix of full length hydrozoan Brachyury proteins.

Multiple sequence alignment of the deduced full-length amino acid sequences of *D. pumila* Brachyury proteins revealed that their T-boxes show about 70–77% identity. By contrast, full-length sequences have an overall lower amino acid identity, thus, the remaining regions are less conserved (Fig. 2a, b).

Further, we performed a multiple sequence alignment of deduced Brachyury proteins, functional domain prediction by *hmmscan*, and a search of the R1 repressor domain with a sequence of R1 from *H. vulgaris* Brachyury1 as query^33^. Hydrozoan Brachyury1 proteins share higher identities with homologous genes in different species than Brachyury2 and Brachyury3 (Fig. 2c, d). The inter-species Brachyury1 identities were also higher than identities between Brachyury paralogs within the same species (Fig. 2b, d). Only Brachyury1 proteins have the repression R1 fragment in the C-terminal region (Fig. 2c, Supplementary Information Fig. S3). Scyphozoan and cubozoan Brachyury2/3 proteins (Supplementary Information Fig. S3) and hydrozoan Brachyury2 and Brachyury3 proteins (Fig. 2c) have lost it. Inter-species sequence comparison further revealed that of all analyzed hydrozoan Brachyury proteins, Brachyury2 proteins have the shortest sequences positioned N-terminally of the T-box (e.g., 20 amino acid for DpBra2), whereas Brachyury3 proteins are the most diverse in length and amino acid identity among hydrozoans (Fig. 2c, d).

### Brachyury gene expression patterns during embryonic development and in shoots of the *D. pumila* colony

To determine whether the Brachyury paralogs are differently expressed during the development of *D. pumila*, we analyzed the spatiotemporal distribution of their transcripts by whole-mount in situ hybridization.

**Figure 3:**
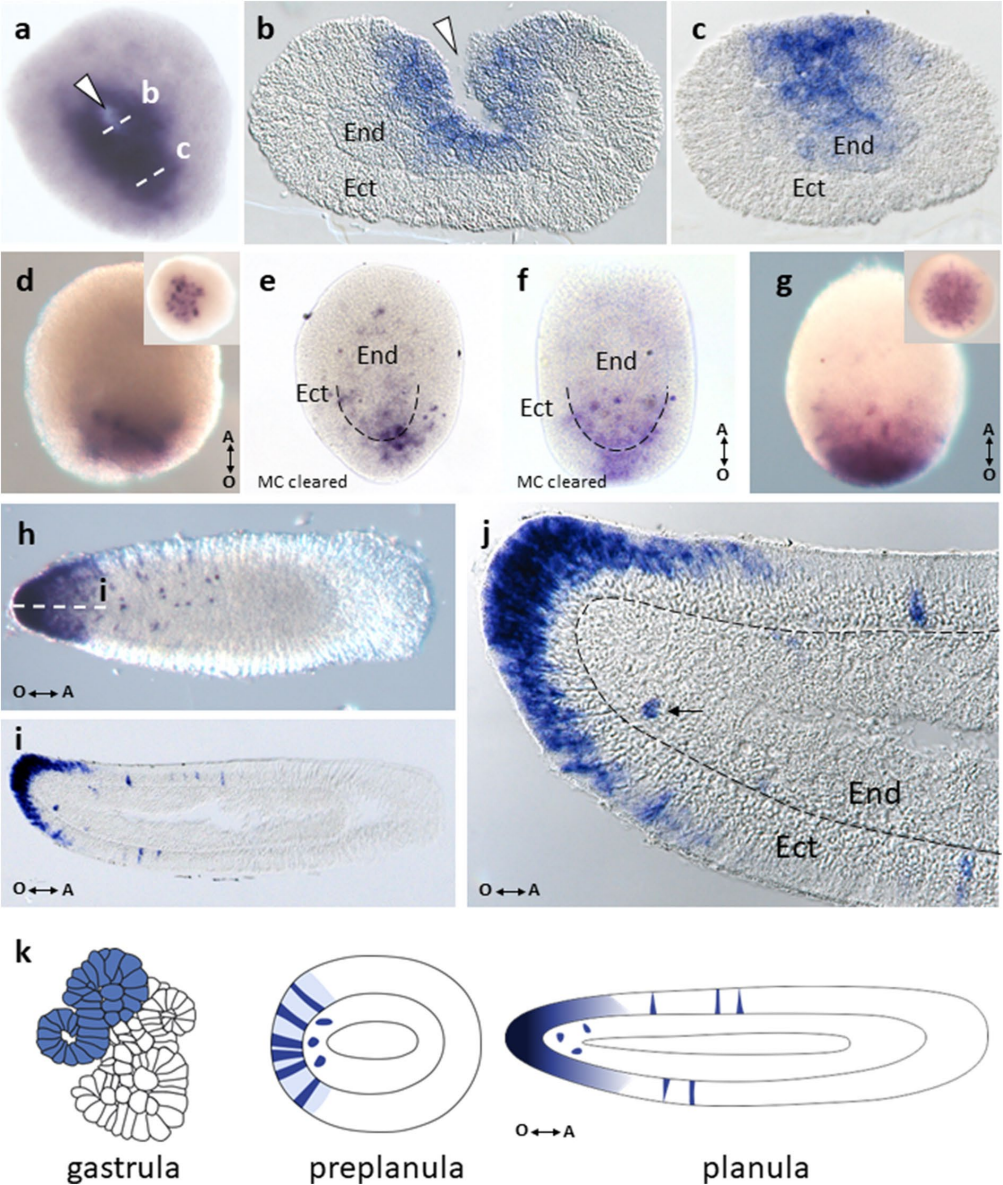
Spatial expression patterns of DpBra1 during embryonic development. (**a**) Expression is apparent in a broad domain at the end of gastrulation. White arrowhead points to the opening in the centre of the toroidal surface. (**b,c**) Transverse sections of the embryo through the levels indicated by the white dotted lines in (**a**). Expression is present both in the ectoderm (Ect) and the endoderm (End). (**d**) Expressing cells are located at the oral pole of the preplanula. Double arrow shows the direction of the oral-aboral (O-A) body axis. (**e**) DpBra1 signal is prominent in the ectoderm and the endoderm of the preplanula cleared with Murray's Clear (MC) solution. (**f,g**) Broad domain of expression biased towards the oral pole in the early planula. (**h**) In the mature planula, expression is observed in the oral end and in individual cells in the oral half of the body. (**i**) Longitudinal section of the planula through the level indicated by the white dotted line in (**h**). Expression is localized in the oral ectoderm almost exclusively. (**j**) A blowup of the (**i**). Black arrow indicates single endodermal cell with DpBra1 expression. Black dotted line marks the basal lamina. (**k**) The scheme of expression patterns of DpBra1 during development.

In *D. pumila*, gastrulation is apolar and mainly proceeds via epithelization of the outer cells. This mode of gastrulation causes deformations of the embryonic surface and results in multiple concavities and indentations. Thus, at the midgastrula stage, multiple epithelized toroidal surfaces compose an embryonic surface. These deformations are smoothed out towards the end of gastrulation, when only several indentations are still visible. The last indentation tends to be located in the oral domain of the embryo. However, this last indentation is not homologous to a blastopore^39^. At the end of gastrulation, in situ hybridization revealed expression of DpBra1 in a unitary broad domain (Fig. 3a) which did not overlap with any specific region within the gastrula stage embryo. Signal was visualized both in the ectoderm and the endoderm (Fig. 3b, c). At the preplanula stage, expression signal was detected in discrete patches both in the ectoderm and the endoderm mostly at the oral end of the embryo (Fig. 3d, e). In the early planula, we observed DpBra1 expression in the oral third of the larva (Fig. 3f, g). In the mature planula, DpBra1 was expressed in the oral half of the larva (Fig. 3h, i). In the ectoderm, we observed two domains of DpBra1 expression. In the tip, biased towards the oral pole expression signal was visualized in apical domains of ectodermal cells. Also, DpBra1 expression was visible in scattered ectodermal cells in the middle of the larva. In the endoderm, expression was present in only a few cells at the oral end (Fig. 3j). Figure 3k represents expression patterns of DpBra1 during development.

**Figure 4:**
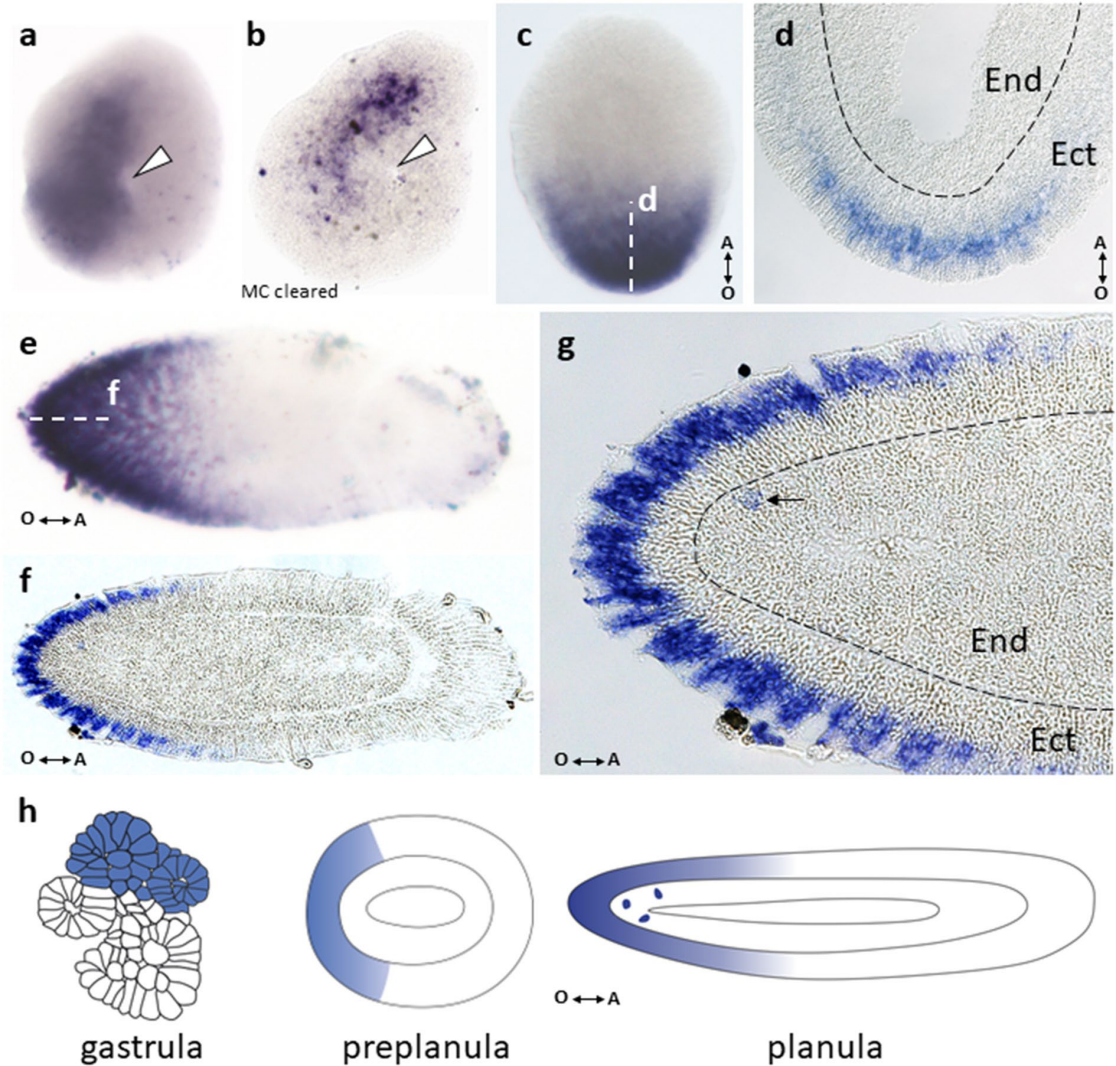
Spatial expression patterns of DpBra2 during embryonic development. (**a**) Broad expression domain at the end of gastrulation. White arrowhead points to the opening in the centre of the toroidal surface. (**b**) Gastrula cleared with Murray's Clear (MC) solution. Expression signal is detected in inner cells of the embryo. (**c**) Biased toward the oral pole expression covers half of the preplanula/early planula. Double arrow shows the direction of the oral–aboral (O–A) body axis. (**d**) Longitudinal section of the embryo through the level indicated by the white dotted line in (**c**). DpBra2 transcripts are visualized in the perinuclear cytoplasm of ectodermal (Ect) cells. *End *endoderm. Black dotted line shows the basal lamina. (**e**) Oral expression is slightly biased to the pole in the mature planula. (**f**) Longitudinal section of the planula through the level indicated by the white dotted line in (**e**). Expression is localized mostly in the oral ectoderm. (**g**) A blowup of the (**f**). DpBra2 transcripts are visualized in apical domains of ectodermal cells. Black arrow indicates single endodermal cell with DpBra2 expression. Black dotted line marks the basal lamina. (**h**) The scheme of expression patterns of DpBra2 during development.

DpBra2 expression was detected both in the ectoderm and the endoderm at the end of gastrulation and also forms a broad domain (Fig. 4a, b). In the preplanula/early planula, DpBra2 expression was observed at the oral half of the embryo with more prominent signal at the oral pole (Fig. 4c). Transcripts concentrated in the perinuclear cytoplasm of ectodermal cells (Fig. 4d). In the mature planula, we observed DpBra2 expression in oral third of the larva with a bias towards the pole (Fig. 4e, f). DpBra2 RNA was visualized in apical domains of ectodermal cells (Fig. 4g). Expression was also present in single endodermal cells (Fig. 4g) as in case of DpBra1 (Fig. 3j). Figure 4h represents expression patterns of DpBra2 during development.

**Figure 5:**
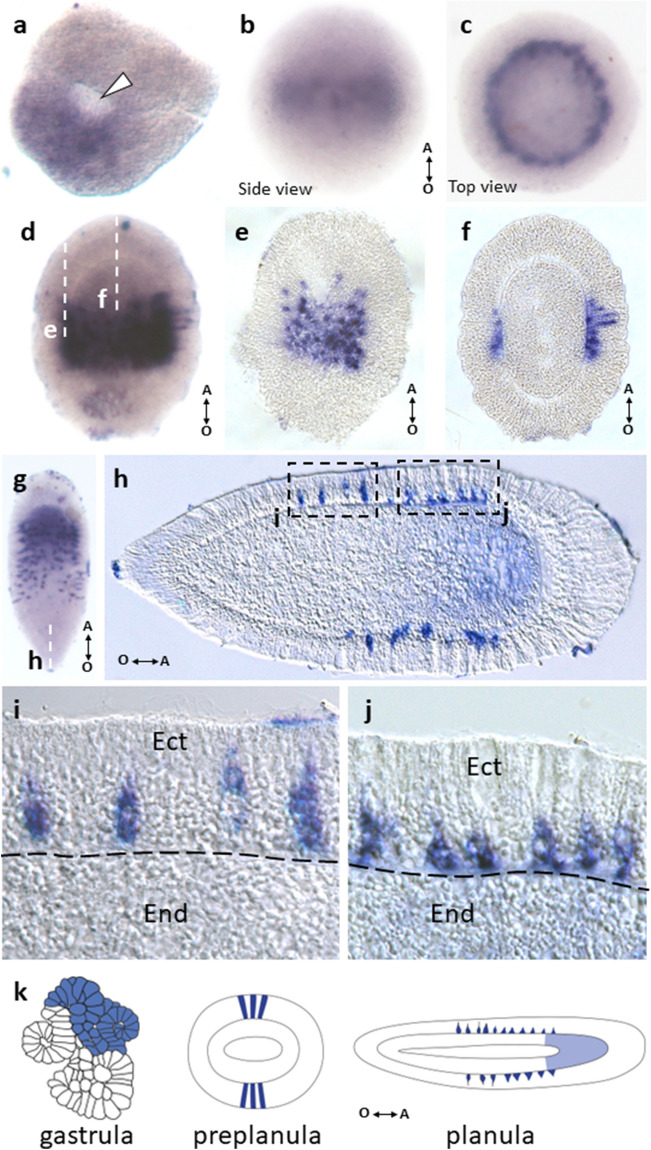
Spatial expression patterns of DpBra3 during embryonic development. (**a**) Broad expression domain at the end of gastrulation. White arrowhead points to the opening in the centre of the toroidal surface. (**b,c**) Expression forms central belt showing in the ectoderm of the preplanula. Double arrow shows the direction of the oral–aboral (O–A) body axis. (**d**) Expressing cells are visible as a broad central belt in the early planula. (**e,f**) Longitudinal sections of the larva through the levels indicated by the white dotted lines in (**d**). Expression is strictly ectodermal, staining is visualized mostly in basal cell domains. (**g**) Intense staining is visible in scattered cells in the central region of the mature larva. Weak staining is observed in the aboral endoderm. (**h**) Longitudinal section of the planula through the level indicated by the white dotted line in (**g**). Intensely stained cells are located in the ectoderm. (**i,j**) Blowups of the (**h**). Columnar and triangular bodies of expressing cells lie right above the basal lamina. *Ect *endoderm, *End *endoderm. Black dotted line marks the basal lamina. (**k**) The scheme of expression patterns of DpBra3 during development.

DpBra3 was expressed in a broad domain at the end of gastrulation (Fig. 5a) in a pattern similar to those of DpBra1 and DpBra2. However, the expression pattern of DpBra3 differed drastically at later developmental stages. At the preplanula stage, DpBra3 signal was visible as a belt in the center of the oral-aboral axis (Fig. 5b, c). As the development proceeded, the expression area expanded to cover the central part of the early planula (Fig. 5d). Longitudinal sections (Fig. 5e, f) revealed that transcripts are present mostly in basal domains of scattered ectodermal cells. As the planula elongated, expression continued in the middle part of the larva in discrete ectodermal cells (Fig. 5g, h). Weak signal also appeared in the aboral endoderm (Fig. 5g, h). Longitudinal sections of the mature planula clearly demonstrated that bottle-like (Fig. 5i) and triangular (Fig. 5j) bodies of DpBra3-expressing cells were located directly above the basal lamina or are between endoderm and ectoderm. The latter probably migrate towards the ectoderm from endoderm (Fig. 5h). Figure 5k represents expression patterns of DpBra3 during development.

**Figure 6:**
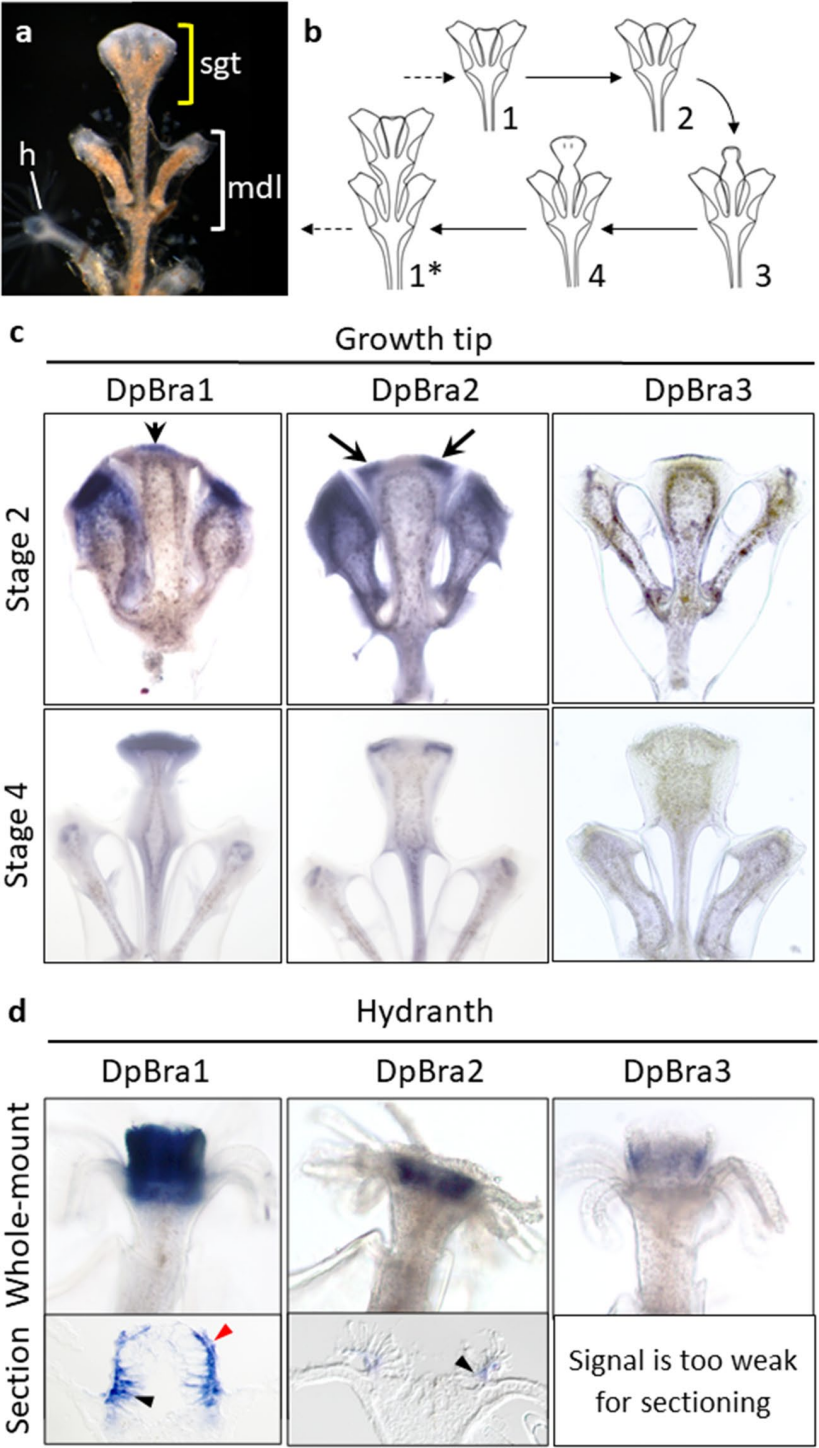
Spatial expression patterns of Brachyury genes in the colony of *D. pumila*. (**a**) The shoot of the *D. pumila* colony. Yellow bracket shows the shoot growth tip (sgt), white bracket—one module (mdl) of the shoot. *h *hydranth. (**b**) The scheme of the morphogenetic cycle in the shoot growth tip of *D. pumila*. Numbers 1–4 indicate successive stages of morphogenesis. After the formation of the new internode, the cycle starts anew (asterisk). (**c**) Spatial expression patterns of Brachyury genes in the shoot growth tips on stage 2 and 4 of the morphogenetic cycle. On the stage 2, DpBra1 expression is apparent in the central apical part of the shoot growth tip. DpBra2 expression is visible at the opposite sides of the shoot growth tip apex. On the stage 4, DpBra1 expression is uniform in the apex. DpBra2 expression remains at the opposite sides of the tip. Arrows point to the areas of expression. Expression of DpBra3 was not detected. (**d**) Spatial expression patterns of Brachyury genes in hydrants (whole-mount and longitudinal section through the center of the hydranth). Expression of Brachyury genes is apparent in the hypostome of the hydranth. Black arrowheads point to expression in the endoderm. Red arrowhead points to expression in the ectoderm.

Further, we examined expression patterns of the three Brachyury genes in the colony shoots of *D. pumila. D. pumila* forms monopodially growing colonies possessing biradial symmetry. Shoots of the colony are composed of repetitive modules. Each module consists of a fragment of the shoot in the center and two hydrants on the sides (Fig. 6a). New modules are formed on the top due to the repeating morphogenetic cycle in the specific organ—the shoot growth tip (Fig. 6b). Stage 1 represents the state when the morphogenetic cycle has not started yet (Fig. 6b1). At stage 2, the growth starts with the apical surface of the tip curving up (Fig. 6b2). At stage 3, the tip elongates and takes a hemispherical shape (Fig. 6b3). At stage 4, the growth tip is dividing into the central and two lateral parts (Fig. 6b4). Lateral primordia further differentiate into hydrants, while the central part will become the new shoot growth tip (Fig. 6b1*).

We analyzed expression patterns of three Brachyury genes in shoot growing tips on stages 2 and 4 of the morphogenetic cycle and in fully formed differentiated hydrants. DpBra1 and DpBra2 expression was detected in the apical ectoderm of the growth tip (Fig. 6c). DpBra1 is expressed in the central part of the apex at stage 2 and uniformly at stage 4. DpBra2 expression was observed in two domains at opposite sides of the apex at stages 2 and 4. Thus, DpBra1 and DpBra2 expression domains do not overlap at stage 2, but are co-expressed at stage 4. DpBra3 expression was not detected in the shoot growth tip.

Expression of three Brachyury genes was observed in the hypostome of the hydranth (Fig. 6d). A longitudinal section through the center of the hydranth revealed that DpBra1 was expressed both in the ecto- and the endoderm. DpBra2 signal was clearly visible in the endoderm of the hypostome, while the presence of a signal in the ectoderm is unclear. Unfortunately, DpBra3 signal was too weak for the fine examination, but seems to be expressed in the ectoderm (when viewed from the surface).

### Brachyury genes are differently regulated by the cWnt signaling in *D. pumila*

**Figure 7:**
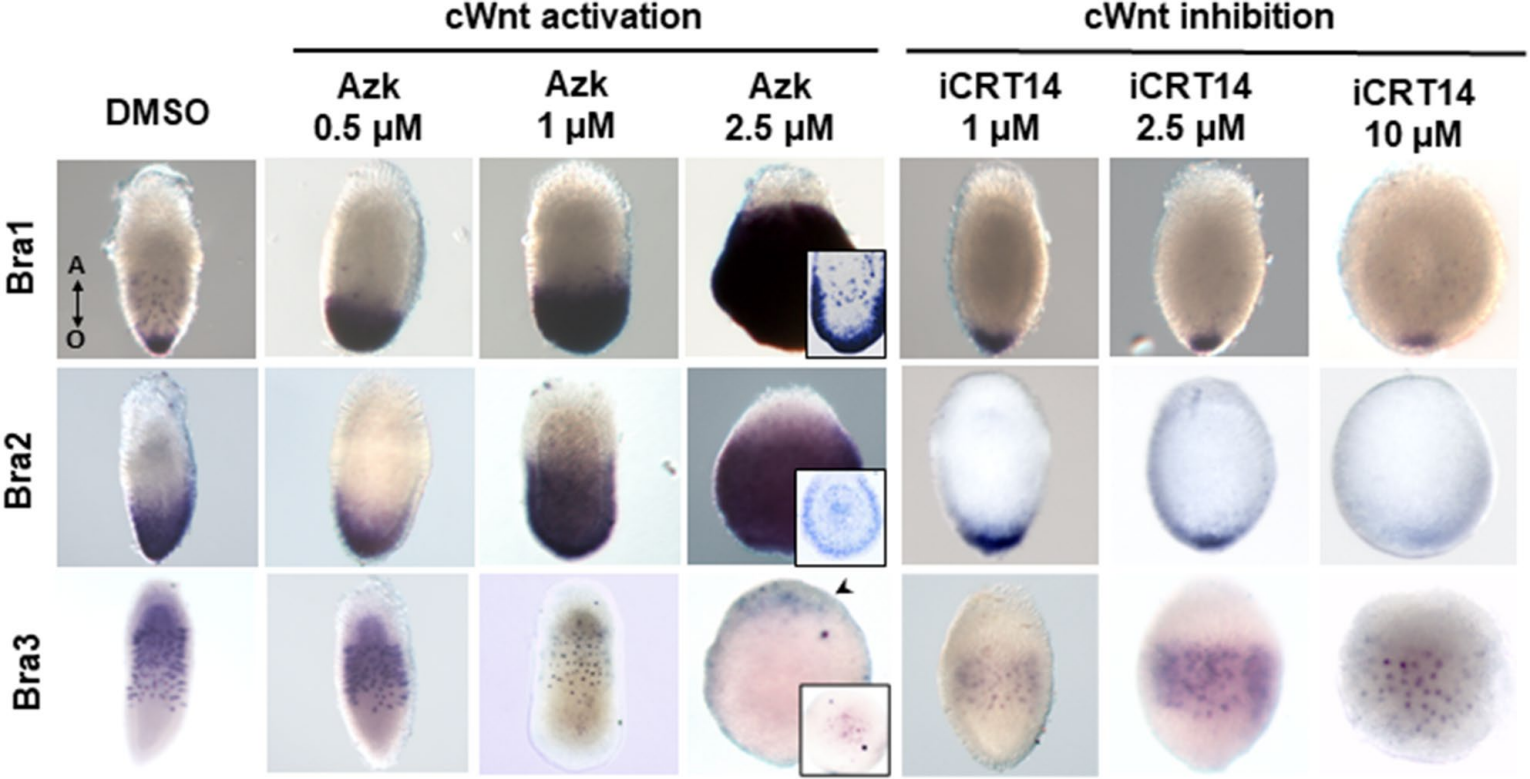
DpBra1 and DpBra2, but not DpBra3, expression depend on the activity of the cWnt signaling pathway. Pharmacological modulations of the cWnt pathway change the area of DpBra1 and DpBra2 expression in planula larvae. Number of endodermal signal-positive cells also increases (see details for longitudinal sections). Hyperactivation of the cWnt results in DpBra3 expression domain shifted aborally. cWnt inhibition does not affect DpBra3 expression notably. Arrowhead points to DpBra3-expressing cells on the oral pole of the larva (see detail). Double arrow shows the direction of the oral–aboral (O–A) body axis for all larvae.

It was shown previously in *Hydra* and *C. hemisphaerica* that two hydrozoan Brachyury genes, Brachyury1 and Brachyury2, are regulated by cWnt signaling^13,33,40,41^. However, it is unknown if Brachyury3 is still a cWnt-dependent gene after the duplication event. We assayed the dependence of three Brachyury genes on the cWnt pathway in *D. pumila*. We treated embryos at the gastrula stage with different concentrations of pharmacological agents to modulate the cWnt pathway, cultivated them until planula stage, and examined then expression patterns of three Brachyury genes in planula larvae of *D. pumila* (Fig. 7). Azakenpaullone (Azk) activates cWnt signaling and iCRT14 inhibits it^42–44^. It was shown in a previous study that hyper-activation of cWnt signaling results in the enlargement of larval oral domain, while its inhibition leads to reduction of oral domain in *D. pumila*^39^.

DMSO-treated (control) larvae had normal morphology and expression patterns of three Brachyury genes (Fig. 7). Treatments with the increasing concentrations of Azk resulted in the gradual expansion of DpBra1 and DpBra2 expression domains. After 2.5 μM Azk treatment, DpBra1 and DpBra2 expression signals were observed in the entire larva except the aboral-most region. The number of endodermal DpBra1- and Bra2-positive cells also increased. Vice versa, gradual inhibition of the cWnt signaling with iCRT14 led to the decrease of DpBra1 and DpBra2 expression domains in area (Fig. 7).

Strikingly, overactivation of the cWnt signaling did not lead to the expansion of DpBra3 expression domain. The belt of DpBra3-expressing cells shifted in more and more aboral positions, vacating the central domain. In the result of 2.5 μM Azk treatment, several DpBra3-expressing cells were detected at the aboral pole of the larva (Fig. 7: arrowhead). Inhibition of the cWnt signaling did not notably change the DpBra3 expression domain (Fig. 7).

### *D. pumila* Brachyury genes differently regulate tissue differentiation in the animal cap assay

To uncover functional differences of three *D. pumila* Brachyury genes, we employed the *Xenopus laevis* animal cap assay system. Using this assay, we surveyed DpBra1, DpBra2, and DpBra3 for their ability to affect cell fates of naive *Xenopus* animal cap cells. It is known, that untreated animal caps differentiate into epidermal tissue^45^, but the injection with *Xenopus* Bra or *Hydra* Bra1 mRNA promotes mesoderm specification, and *Hydra* Bra2 mRNA shows neural-inducing activity^33,46^. We injected capped mRNAs encoding DpBra1, DpBra2, or DpBra3 into the animal region of two- to four-cell stage embryos (~ 1 ng per embryo), dissected the animal caps at the blastula stage (stage 8), cultured them until control embryos reached late neurula stage (stage 18), and examined marker gene expression in these caps using conventional or quantitative RT-PCR (qRT-PCR). Uninjected animal caps were used as a control group.

**Figure 8:**
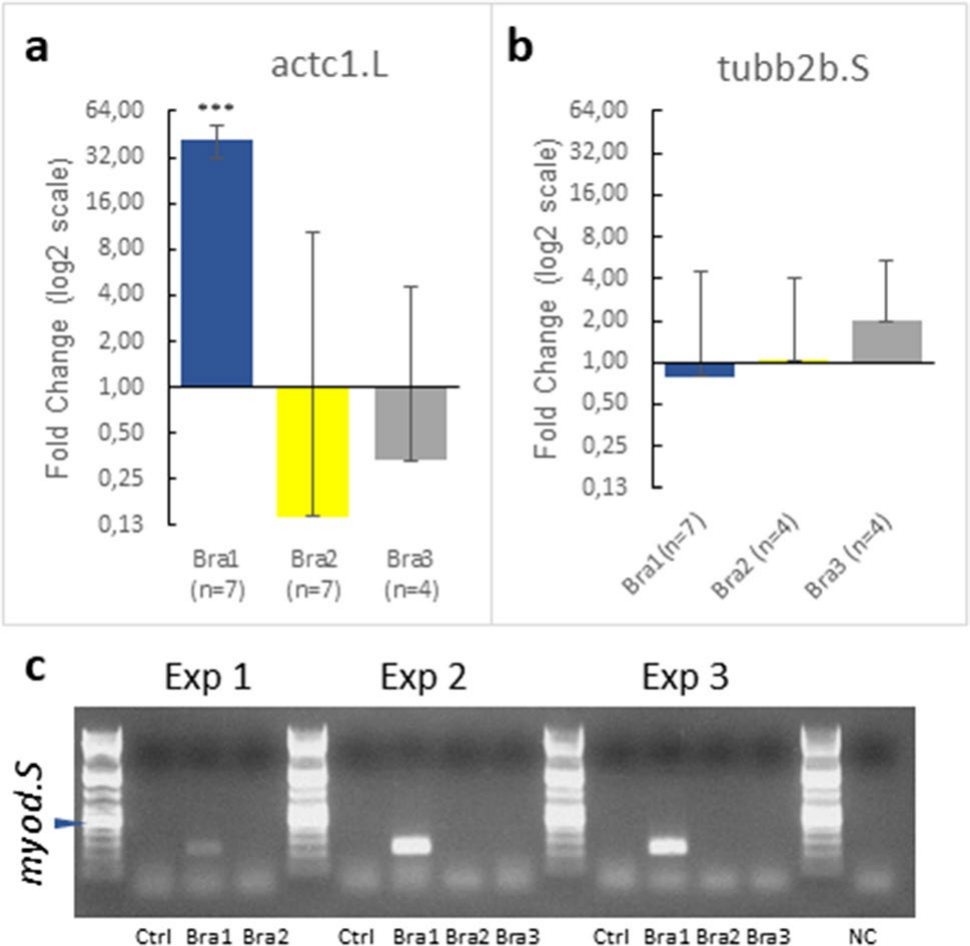
Molecular phenotype of *Xenopus* animal caps injected with *D. pumila* Brachyury genes. (**a,b**) RT-qPCR analysis on the induction of actc1.L, myod2.S, and tubb2b.S by *D. pumila* Brachyury mRNAs. Data are presented as normalized fold change expression (mean ± s.d.) in experimental groups. *n *n-value. The number of experimental groups is 3, 3, and 2 in (**a**); 3, 2, and 2 in (**b**). ***p < 0.001. (**c**) Gel electrophoresis for myod.S after injection by *D. pumila* Brachyury mRNAs. *NC *negative control. *L* DNA ladder. Arrowhead points to 500 bp band of the DNA ladder. See non-processed gel image in Supplementary Information Fig. S4.

DpBra1 significantly (P < 0.0001) induced the expression of mesodermal marker gene actc1.L (muscle actin)^47^ (Fig. 8a) as well the expression of another mesodermal marker gene, myod.S^47^. Since myod.S expression was too low for reliable quantification in the control group using qRT-PCR, we used gel electrophoresis to show the induction (Fig. 8c). DpBra1 did not affect the expression of neural marker gene tubb2b.S^47^ (Fig. 8b) while DpBra2 and DpBra3 did not affect the expression of neuronal and mesodermal marker genes (Fig. 8).

## Discussion

Gene duplications facilitate the evolution of regulatory genes, driving expansion of families of signaling molecules and transcription factors^48,49^. Duplication event produces two copies (paralogs) of a gene both of which are orthologous to the “parent” gene. Subsequent evolution may lead to divergence: one copy retains high similarity to its orthologs while the second undergoes structural changes and may be designated as a daughter or child copy^50^. In the present study, three paralogous genes of Brachyury transcription factor were found in hydrozoan lineage. The phylogenetic reconstruction suggests the first duplication event occurred before the hydrozoan clade branched off (in the common ancestor of meduzosoans or earlier). The “daughter” copy of the first duplication (Brachyury2/3) experienced then an additional duplication event in the common ancestor of hydrozoans (Fig. 1).

There are three main scenarios after gene duplication. First outcome leads to loss of function of duplicated copy which becomes a pseudogene or is lost. Two other outcomes are subfunctionalization and neofunctionalization^51,52^. In case of subfunctionalization, two duplicated copies share the original function of the ancestral gene, and both are required to preserve the entire ancestral function^51,53^. Overlapping expression domains of two duplicates often reflect occurred subfunctionalization^51^. In case of neofunctionalization, one copy retains its ancestral functions, and the other one is free to gain a novel function, since it is relaxed from the selective pressure^53^, and often acquires the expression domain different from the ancestral gene^54,55^. The acquisition of novel functions by regulatory genes plays a key role in diversification of developmental pathways and body plans in metazoans^56,57^. Importantly, the same duplicate can also display both features of sub- and neofunctionalization with regard to different functions^58^.

Evolution of Brachyury reveals signs of both sub- and neofunctionalization. In teleosts, expression of two Brachyury genes reveals the common chordate pattern^28,59^. Only simultaneous loss of both Brachyury genes recapitulates mouse homozygous brachyury mutant phenotype^4,28^, indicating subfunctionalization followed duplication of Brachyury in teleosts. On the contrary, neofunctionalization seems to follow Brachyury duplication in *X. laevis* as XBra3 (tbxt2.L/tbxt2.S) is distinct from XBra and XBra2 (tbxt,L/tbxt,S) in function and spatio-temporal pattern of expression^60^. Within hydrozoans, outcomes of Brachyury duplication previously were studied in *Hydra*^33^. Though both Brachyury paralogs are expressed in the hypostome of *Hydra*, they evolved distinct coding sequences and diverged their functions. Authors posit, that Brachyury paralogs show a mixture of sub- and neofunctionalisation in *Hydra*^33^. However, it is unknown, if similar outcomes have shaped the roles of Brachyury genes in other cnidarians.

Brachyury genes are involved in axial patterning also in other studied cnidarians ^14^. In cnidarian species with polar gastrulation, where it is linked to the axial patterning and occurs in the oral region of the embryo^61^, Brachyury expression accompanies gastrulation morphogenetic movements^12,19,20,36^. In gastrulating *C. hemisphaerica,* Brachyury paralogs (ChBra1 and ChBra2) display overlapping patterns^19^ and are important for progression of gastrulation^13^. In *D. pumila* during gastrulation, all three Brachyury paralogs are expressed in a broad domain (Figs. 3a, 4a, 5a). In contrast to the cnidarian species with polar gastrulation^12^, it is unlikely, that Brachyury genes provide demarcation of ecto-endoderm boundary in *D. pumila*, since germ layers specification is not associated with axial polarity and oral region in particular during gastrulation in this species^39^.

Predominantly oral expression of Brachyury genes continues throughout a cnidarian life cycle. Brachyury is expressed in pharynxes of anthozoan larvae^12,20^ and in the oral ectoderm of hydrozoan larvae in *C. hemisphaerica* and *D. pumila*, where expression of Brachyury1 and Brachyury2 is detected^19^ (Figs. 3h–j, 4e–g). Surprisingly, Brachyury3 orthologs are not associated with oral tissues in hydrozoan larvae. Previously examined Brachyury ortholog of *P. carnea* which is expressed in the aboral ectoderm^36^ clusters according to our analysis with Brachyury3 group (Fig. 1). In *D. pumila*, Brachyury3 displays expression in discrete triangular and bottle-like ectodermal cells (Fig. 5g–j), morphologically similar to sensory cells of cnidarian nervous system^62^, though Brachyury is known to act as neural repressor in the anthozoan *Nematostella*^14^. Moreover, DpBra3 seems not to be a cWnt-dependent which is in contrast to reported Wnt-dependence of Brachyury expression in cnidarians and bilaterians ^14,63^ including DpBra1 and DpBra2 (Fig. 6). Brachury3 orthologs display strong diversity in length and amino acid identity among hydrozoan Brachyury gene family (Fig. 2c, d). Differences in protein sequences, regulation, and expression domains suggest that the newly derived Brachyury3 diverged to different functions in hydrozoan species.

In the hydranth of *D. pumila*, DpBra3 expression pattern does not drastically differ from patterns of DpBra1 and DpBra2 and is in line with previous studies^33,36,64,65^. All three Brachyury paralogs were detected in hypostome of hydranth with overlapping patterns (Fig. 6d). Likely, the original function of Brachyury in a hydrozoan hydranth is associated with a specification of an oral domain (a hypostome) as a whole. As in *Hydra*^33^, overlapping expression domains of Brachyury paralogues in hypostomes of *D. pumila* hydranths suggest occurred subfunctionalization^51^.

In the shoot growing tip of *D. pumila* colonies^66^, DpBra1 and DpBra2 are strongly expressed in its apical ectoderm (Fig. 6c), which could be considered a derivative of the larval oral domain. Association of DpBra2 expression with the formation of hydranth primordia indicates its novel function in the hydranth primordia in *D. pumila*.

The specificity of Brachyury function is mostly defined by the N- and C-terminal domains, but not by the central T-box^9,67^. In line with previous studies^33^ and our data (Fig. 8), high functional conservation of hydrozoan Brachyury1 orthologs is consistent with high conservation of protein sequence (Fig. 2c). However, our data indicates the functional divergence of Brachyury2 and Brachyury3. In *Xenopus* animal cap assays, DpBra2 and DpBra3 did not cause increased expression of the mesodermal markers actc1.L or myod.S (Fig. 8a, c). In Brachyury2 and Brachyury3, N- and C-terminal domains show lesser amino acid identity to the ancestral gene and have lost ancestral C-terminal repression domain R1 (Fig. 2a–c). These differences in terminal domains could be responsible for the neo- and subfunctionalization of Brachyury2 and Brachyury3 in hydrozoans, even though it was suggested that they occur mainly due to mutations in regulatory sequences, rather than mutations in the coding sequence^68^.

Taken together, our data indicate two duplication events of Brachyury in cnidarians. Brachyury1 is the most conservative duplicate, both on the functional and sequence levels. In studied hydrozoans and in *D. pumila* in particular, it is supposed to preserve its ancestral function as a crucial component of axis formation and patterning. Hydrozoan Brachyury 2 and Brachyury 3 reveal features of sub- and neofunctionalization. Brachyury3 however, displays strong divergence in sequence and functions among hydrozoans. Our data on Brachyury support the model of an indistinct border between sub- and neofunctionalization and complex outcomes for duplicated genes ^58^, and provides a promising model for studies on post-duplication scenarios.

## Methods

### Animals and sampling

Sampling of *D. pumila* colonies and experimental procedures over *D. pumila* embryos were performed at the Pertsov White Sea Biological Station (Lomonosov Moscow State University) (Kandalaksha Bay; 66°340 N, 33°080 E) during the period of *D. pumila* sexual reproduction (June–July). Sexually mature colonies were kept in natural seawater at + 10–12 °C. Whole-mount observations were made under a stereomicroscope Leica M165C.

### Chemical treatment

To activate/inhibit cWnt signaling, gastrulating embryos were treated with 0.5/1/2.5 μM 1-Azakenpaullone (Sigma, Canada/China) or 1/2.5/10 μM iCRT-14 (Sigma, USA/China) respectively. Stock solutions were prepared with DMSO at 10 mM, aliquoted and stored at − 20 °C. Working solutions were prepared before use by dilution of stock solutions in filtered seawater (FSW) to the final concentration. Control embryos were exposed to 0.1% DMSO in FSW. Working solutions were refreshed daily. Incubation was performed in the dark.

### Data sources and transcriptome assembly

To analyse phylogenetic relationships within the brachyury gene family, we surveyed 28 metazoan species. Gene sequences were obtained from several sources (Supplementary Information Table S1). Bilaterian, ctenophore, placozoan and anthozoan sequences were obtained from nucleotide collection of NCBI database. Some assembled cnidarian transcriptomes were downloaded from public databases at NCBI (*Aurelia aurita*, *Morbakka virulenta*, *Nemopilema nomurai*, *Podocoryna carnea*, *Lucernaria quadricornis*, *Tripedalia cystophora*^69^, *Dynamena pumila*^35^, *Polypodium hydriforme*^70^ or other web-sites (*Clytia hemisphaerica*^71^, *Hydractinia symbiolongicarpus*^72^), *Hydra*^38^). Transcriptomes of *Craspedacusta sowerbii* and *Margelopsis haeckelii* were newly assembled by ourself. Data for *Margelopsis haeckelii* were collected and sequenced de novo and are available in our lab. Read quality control was performed with fastp (v.0.20.0) software^73^. De novo transcriptomes were assembled with rnaSPAdes (v.3.13.1)^74^ software. Quality of assembly was assessed using BUSCO v.3.0.2 with metazoan database^75^.

### Phylogenetic analyses

Brachyury genes ABJ16449.1 and JAC85032.1 of *C. hemisphaerica* were used as queries for local tblastx search of Brachyury genes in *D. pumila* transcriptome. Using three obtained sequences of *D. pumila* Brachyury-like genes as queries, we searched for Brachyury-like genes in ten other medusozoan transcriptomes. We surveyed 12 medusozoan transcriptomes in total (Supplementary Information Table S1). We also used sequences of bilaterian, ctenophore, placozoan and anthozoan Brachyury genes.

Nucleotide sequences with no corresponding protein sequence in the NCBI database were translated using Transdecoder v5.5.0. The search of T-boxes in analyzed sequences was performed with NCBI Conserved Domain Search tool. Amino acid sequence alignments and phylogenetic analysis were performed with MUSCLE algorithm in MUSCLE software (v3.8.31)^76^. Sequences of Tbx genes were selected as an outgroup. Sequence alignments were trimmed by removing poorly aligned regions using TrimAL tool, v.1.2rev59^77^. A heuristic approach “automated1” was used to select the best automatic method to trim our alignments. Trimming was performed without manual adjustment. Phylogenetic analysis was performed with Maximum Likelihood using IQTree v.2.0-rc2 software^78^. The JTT + R5 model was found to be optimal. To assess branch supports, bootstrap values were calculated running 1000 replicates using ultrafast bootstrap (UFBoot)^79^. Trees were visualized in FigTree v1.4.4 software. Obtained phylogenetic trees were processed with Adobe Illustrator CC. No corrections were made to the tree topology and the branch lengths.

We searched for Brachyury transcripts in gene models of Hydra 2.0 and HydraAEP genome assemblies using phylogenetically-informed annotation pipeline PIA3^80^. PIA3 pipeline is modified from PIA2^81^ and is available on GitHub^82^. Modifications allowed us to automatically retrieve T-box protein class information.

To analyze functional domains of the hydrozoan Brachyury, selected protein sequences were scanned against Pfam hidden Markov model (HMM) database using *hmmscan* of HmmerWeb v.2.41.1^83^. Identification of the conserved R1 domain within the hydrozoan Brachyury was carried out using ClustalW sequence alignment service^84^ with the R1 domain in HyBra1^33^ as a query. The domain architecture of proteins was visualized using Pfam^85^. Multiple sequence alignment and calculation of the identity matrix of hydrozoan Brachyury proteins and T-boxes of *D. pumila* Brachyury were conducted using ClustalW with default settings and shaded using BOXSHADE 3.21.

### *D. pumila* genes isolation, PCR, and antisense RNA probe synthesis

cDNA expression library was prepared by the SMART approach from total embryonic RNA with a Mint cDNA synthesis kit (Evrogen, Russia). cDNA gene fragments were isolated from the library by PCR with gene-specific primers (see Table 1). Primers were designed based upon sequences obtained from the sequenced transcriptome (Illumina) of *D. pumila*^35^. Amplified fragments were cloned into the pAL-TA vector (Evrogen, Russia). Digoxygenine‐labeled antisense RNA probes were generated from gene fragments, which were amplified from plasmids with *D. pumila* genes.

**Table 1:** PCR and qPCR primers used in this study.

Gene	Direct primer 5' ‑> 3'	Reverse primer 5' ‑> 3'
DpBra1 in situ probe	TTGGTGGCGACAGCGAAGAA	CGGCCACGTGTTGTTTTGAATG
DpBra2 in situ probe	GAACGGAGAGGGCAAAGACAAAC	GACGGCGAATATGGGGAACAAAT
DpBra3 in situ probe	AATAATTCTTCACCGTCCAACAGG	CGCGCTTTTCGTGATAGATAGG
XlTubb2b.S (β-tubulin) qPCR	GATCCTACCGGCAGTTACCA	TGACAGAGTCCATTGTGCCT
XlActc1.L (cardiac actin) qPCR	CTATGTGGCTTTGGACTTTGAG	GCTGTTGTAGGTAGTTTCATG GA
XlMyod1.S qPCR	AGTGACAGCCCAAATGACTC	AGAAGGGATGGTGATTACTCTC
XlEF1a qPCR	CCCTGCTGGAAGCTCTTGAC	GGACACCAGTCTCCACACGA
XlODC qPCR	GGGCTGGATCGTATCGTAGA	TGCCAGTGTGGTCTTGACAT

### In situ hybridization

The in situ hybridization protocol was performed as previously described in ^66^ for *D. pumila* shoots and hydranths and in ^39^ for *D. pumila* embryos. An urea-based in situ hybridization method was used for the hydranths^86^.

Shoots were fixed with 0.2% glutaraldehyde/4%formaldehyde in FSW for 1 min and then for an additional hour with 4% formaldehyde in FSW. Samples were washed with PTw (1× PBS with 0.1% Tween 20) thrice and stored in 100% methanol no more than overnight at −20 °C until hybridization. Embryos were fixed with 4% paraformaldehyde in FSW overnight at + 4 °C, rinsed with PBS, and stored at −20 °C in 100% methanol until hybridization.

Samples were rehydrated with PTw and treated with proteinase K (80 μg/ml, 22 °C) for 1–3 min. To inactivate the endogenous alkaline phosphatase and avoid a false positive result, samples were heated at + 80 °C for 30 min. Hybridization was performed at 62 °C (shoots) or 58 °C (embryos) with digoxigenin-labelled antisense RNA probes (1 ng/μL). Anti‐DIG alkaline phosphatase-conjugated antibody (Roche; 1/2000 diluted) and NBT/BCIP substrate (Roche) were used to detect the probe. Stained samples were washed with PTw and methanol to reduce background staining and mounted in glycerol (87%).

Several specimens were treated with Murray's Clear solution (2:1 mixture of benzyl benzoate and benzyl alcohol) to achieve optical tissue clearing. Several specimens were embedded into Technovit resin. Sections (5–7 μm thick) were cut using Reichert-Jung (Leica) Ultra-cut 701701 ultramicrotome (Reichert-Jung, Austria). Imaging of samples was conducted using Leica M165C microscope (Leica, German) equipped with Leica DFC420C (5.0MP) digital camera.

### Animal cap assay

Wild-type *Xenopus laevis* were obtained from the European Xenopus Resource Centre (EXRC) at University of Portsmouth, School of Biological Sciences, UK, or Xenopus 1, USA. Frog maintenance and care was conducted according to standard procedures in the AquaCore facility, University Freiburg, Medical Center (RI_00544) and based on recommendations provided by the international Xenopus community resource centers NXR and EXRC as well as by Xenbase (http://www.xenbase.org/, RRID:SCR_003280). This work was done in compliance with German animal protection laws and was approved under Registrier-Nr. G-18/76 by the state of Baden-Württemberg.

*X. laevis* eggs were collected and in vitro-fertilized, then cultured and microinjected by standard procedures^87^. Embryos were injected two times/embryo with mRNAs at two-cell or four-cell stage using a PicoSpritzer setup in 1/3× Modified Frog Ringer’s solution (MR) with 2.5% Ficoll PM 400 (GE Healthcare, #17-0300-50), and were transferred after injection into 1/3× MR containing Gentamycin. Drop size was calibrated to about 7–8 nL per injection. Injected or uninjected (control) embryos were cultured until st. 8. Animal caps were dissected in 1× Modified Barth’s solution (MBS) and transferred to 0.5× MBS + Gentamycin. 10–15 organoids were collected in TRIzol per condition and experiment.

Full-length *D. pumila* Brachyury sequences were amplified from cDNA library and cloned into pCS2 + 8 plasmid. pCS2 + 8 was a gift from Amro Hamdoun (Addgene plasmid #34931; http://n2t.net/addgene:34931; RRID:Addgene_34931)^88^. mRNAs were prepared using the Ambion mMessage Machine kit using Sp6 (#AM1340) supplemented with RNAse Inhibitor (Promega #N251B) after plasmid linearization with Not1, and injected at 50 ng/μl.

### RT-PCR

Total RNA was extracted using a standard Trizol (Invitrogen #15596026) protocol and used for cDNA synthesis with either iScript cDNA Synthesis Kit (Bio-Rad #1708891). qPCR-reactions were conducted using Sso Advanced Universal SYBR Green Supermix (Bio-Rad #172-5275) on a CFX Connect Real-Time System (Bio-Rad) in 96-well PCR plates (Brand #781366). Conventional PCR and gel-electrophoresis was conducted analogously on a S1000 Thermal cycler (Bio-Rad). See Table 1 for gene-specific primers.

Expression values were normalized against two housekeeping control genes—EF1 and ODC (2^∆∆Ct^ method). Results are presented as means ± standard deviation (s. d.) of the relative fold change (rFC), which is a ratio of normalized mRNA level of the analyzed gene expression in experimental group in comparison to control group.

### Statistical analysis

Statistical analysis of the normalized gene expression data after qRT-PCR was performed in GraphPad Prism 5 software. Normality of data distribution was checked by the Kolmogorov–Smirnov tests. Differences between groups were assessed with one-way ANOVA followed by Dunnet post hoc test. Significance is indicated by asterisks on the graphs. A P-value less than 0.05 was considered significant for all analysis. All experiments were designed with matched control conditions to enable statistical comparison. The n value is 7 for a control group. The n value for each experimental group is stated on graphs.

### Image processing

Pictures were edited with Adobe Photoshop CS6 programs. To achieve optimal exposure and contrast, alterations to the “Brightness'', “Contrast”, “Exposure”, and “Levels” for the RGB channel were used. All tools were applied to the entire image, not locally.

## Supplementary Information

Supplementary Figures.

Supplementary Table S1.

## Data Availability

Sequences obtained in this study have been deposited in GenBank (OP828770–OP828776, OP902368, OP902367).
