# Supplementary material for: The evolutionary history of Brachyury genes in Hydrozoa involves duplications, divergence, and neofunctionalization
Source: Sci Rep. 2023 Jun 9;13:9382. doi: 10.1038/s41598-023-35979-8 (PMC10256749; doi:10.1038/s41598-023-35979-8)
Supplement: Supplementary file 1 — Supplementary Figures. [file 41598_2023_35979_MOESM1_ESM.pdf]

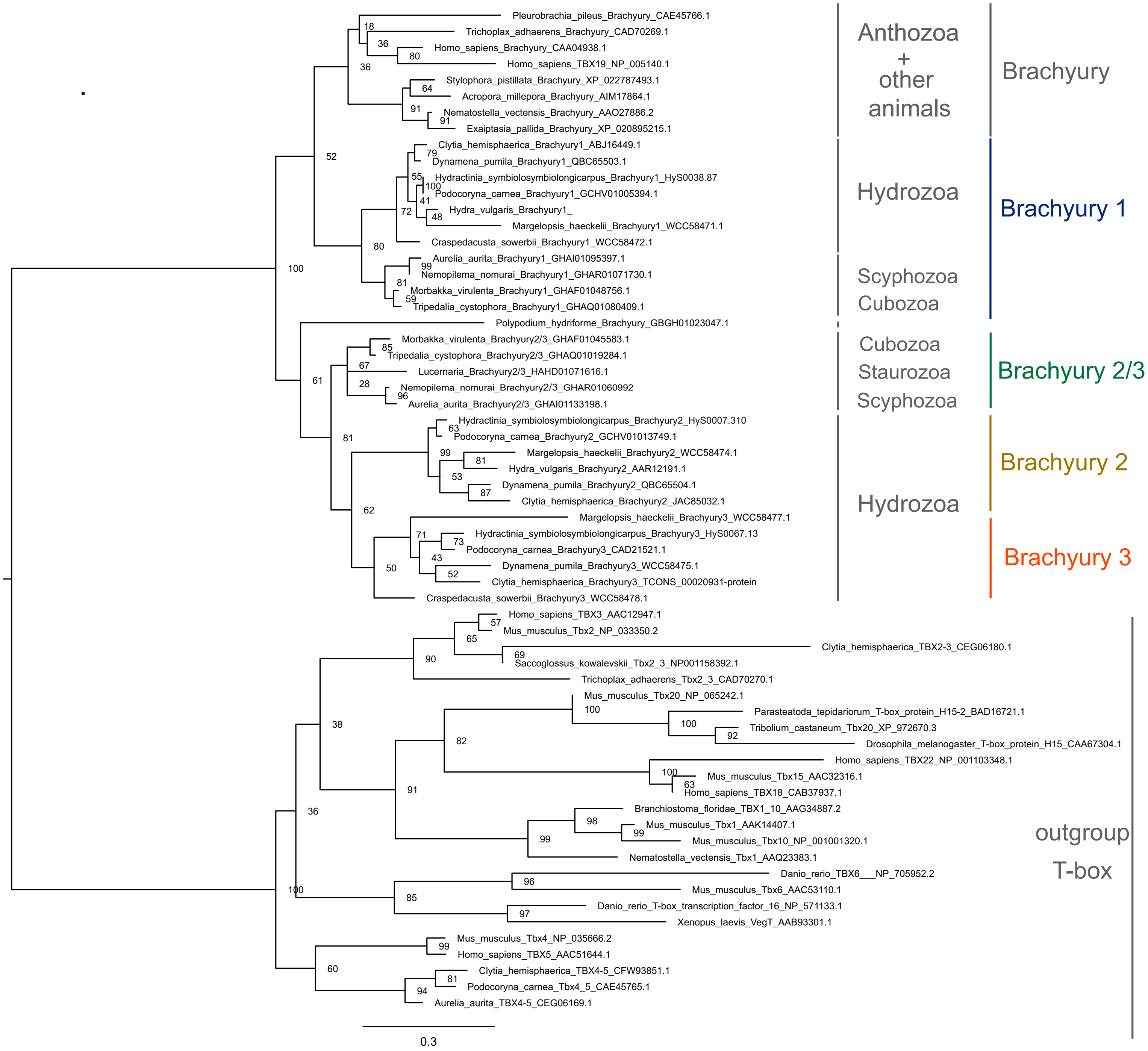

Figure S1. ML phylogenetic tree of T-box domain sequences. Numbers at nodes are bootstrap values, shown as percentages. The scale indicates expected amino acid substitution per site.

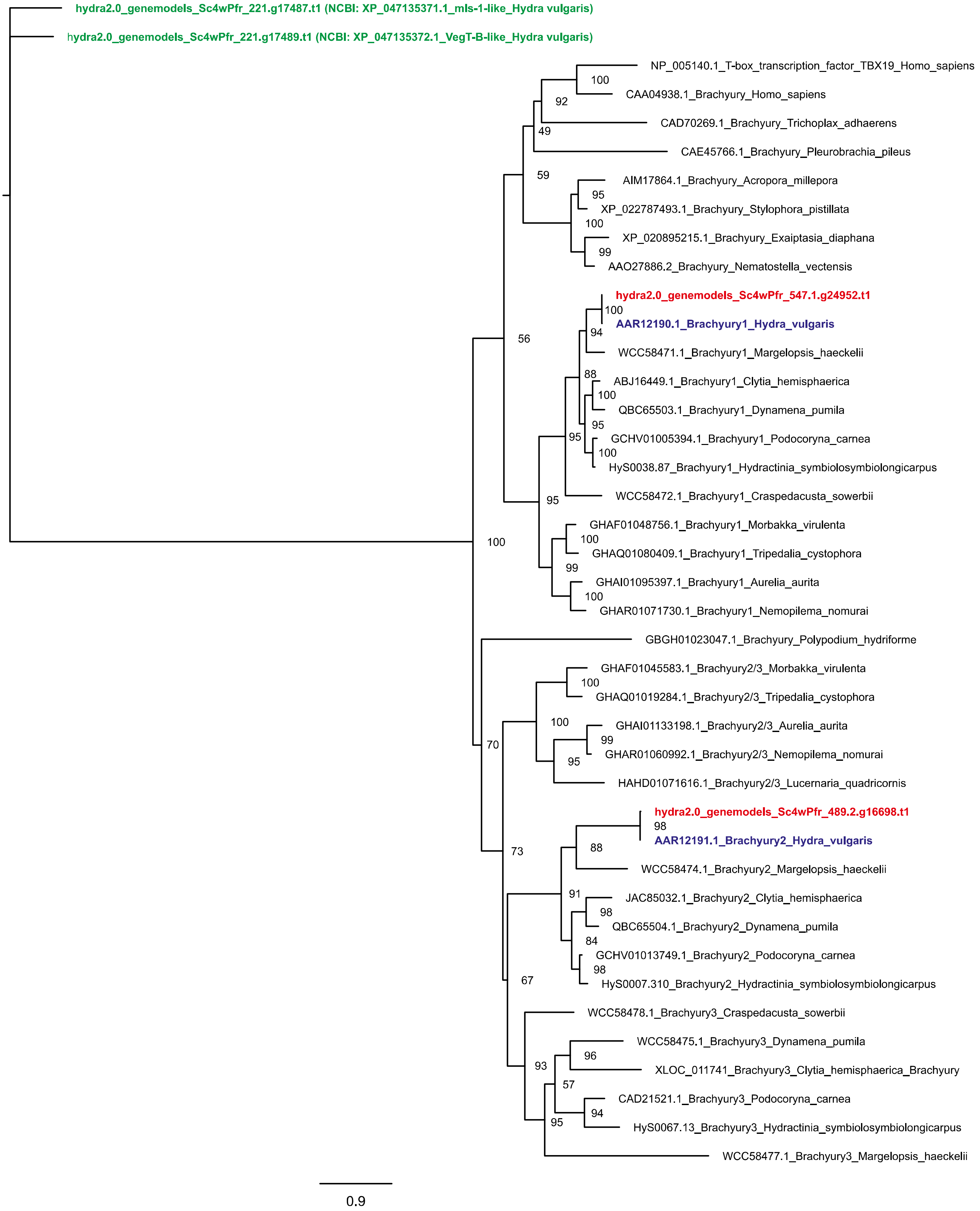

Figure S2. Phylogenetic tree output of automatic search for Brachyury genes in Hydra2.0 genome (https://research.nhgri.nih.gov/hydra/) with PIA3 pipeline. Numbers at nodes are bootstrap values, shown as percentages. The scale indicates expected amino acid substitution per site.

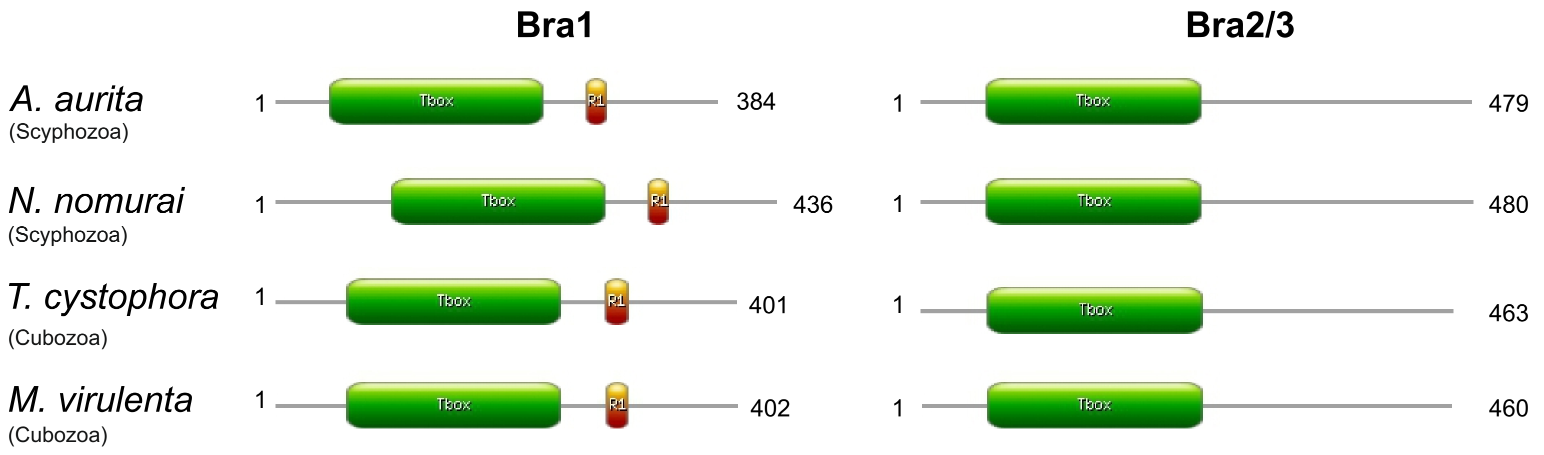

Figure S3. Domain architecture of predicted scyphozoan and cubozoan Brachyury proteins. Green box corresponds to T-box domain, red box corresponds to R1 repressor domain. Numbers indicate the length of the protein in amino acids.

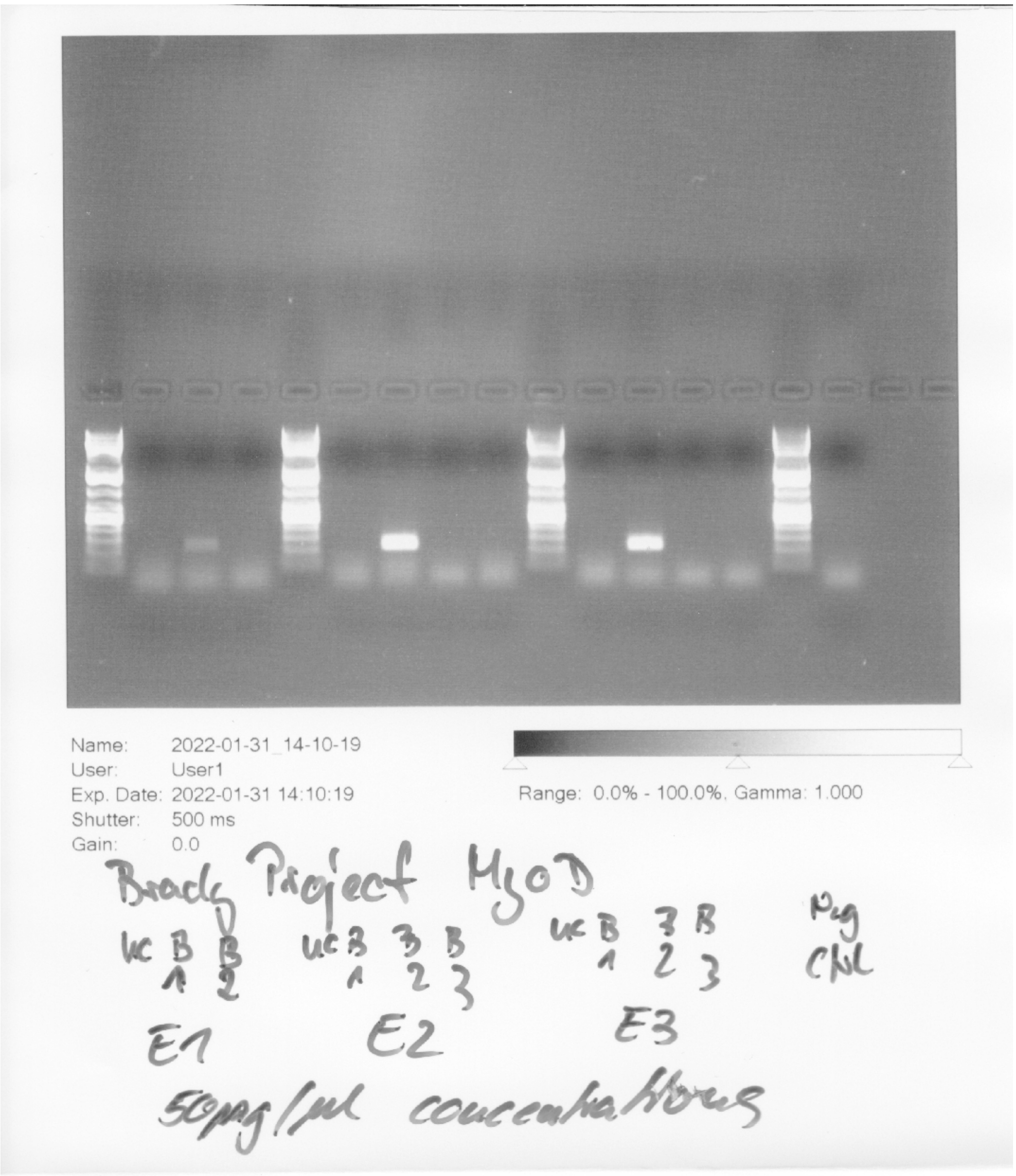

Figure S4. Original non-processed photo of the gel (Figure 8c).
